# For Your Eyes Only: A Field Experiment on Nudging Hygienic Behavior

**DOI:** 10.3389/fpsyg.2020.603440

**Published:** 2020-12-04

**Authors:** Hilde Mobekk, Dag Olav Hessen, Asle Fagerstrøm, Hanne Jacobsen

**Affiliations:** ^1^ Department of Behavioural Sciences, OsloMet – Oslo Metropolitan University, Oslo, Norway; ^2^ Department of Biosciences, University of Oslo, Oslo, Norway; ^3^ Department of Technology, Kristiania University College, Oslo, Norway

**Keywords:** field experiment, nudging, sanitizing, cooperation, observing eyes

## Abstract

These days many gyms and fitness centers are closed to reduce transmission of the SARS-CoV-2 virus in society. The gym is an environment rich in microorganisms, and careful hygiene is a necessity to keep infections at bay. Exercise centers strive for better hygiene compliance among their members. This effort has become essential in light of the current pandemic. Several experimental studies show that others’ physical presence, or the “illusion” of being watched, may alter behavior. This article reports on a natural field experiment testing one specific social nudge intended to increase gym members’ hygienic behavior. The study was conducted before the SARS-COV-2 pandemic. A picture of “observing eyes” was attached to paper dispensers and cleanser spray bottles at two different gyms in Norway. A reversal design, also called an ABA design, with and without the nudge’s presence, was used to investigate the impact on gym members’ hygienic behavior. A follow-up study was conducted in one of the centers to investigate whether the nudge stimuli would function over time. The study included 254 individual choice situations during nine observation sessions conducted over 9 weeks. The results from both centers provide evidence of a strong effect of the nudge. However, the effect decreased during the follow-up study. These findings support previous research indicating that human behavior is influenced by the presence of implicit observation cues – in this case – observing eyes. However, insights into the long-term effect of implicit observation cues are still needed since the salience of the stimuli faded over time.

## Introduction

We are exposed to potential sources of infection every day. The transmission of microorganisms happens through direct and indirect contact with people, animals, and through contact with objects. Many pathogens, such as viruses, bacteria, and fungi, are easily transmitted through close contact, and some can survive in the environment for days (e.g., Institute of Medicine, 2011; Centers for Disease Control and Prevention, 2019). The most significant human death tolls have historically been infectious diseases (Van Bavel et al., 2020). Situations that increase transmission of pathogenic microorganisms include living or working with other people, nursing, sharing items, or visiting public areas. Insufficient hygiene can contribute to elevated infection rates, particularly in pandemic situations (Aiello and Larson, 2002; Curtis and Cairncross, 2003). The risk of infection at premises and meeting places varies with individual and contextual factors, not the least by the type of infectious agent. Interventions to improve hygienic behavior in public places can decrease the transmission of viruses and other infections agents and further increase general health and well-being. Therefore, infection prevention measures should, like vaccines, be a shared responsibility in society.

Exercise and training are important for health and well-being. The numbers of people using gyms and fitness centers have steadily increased over the last decade. By 2019, over 64 million people in the United States were members of one of the 41,000 health clubs in the United States (Statista, 2020). Many people use the workout equipment available in the gyms during the day. Numerous members sweat and are in contact with the exercise equipment with bare skin. Cleaning is an efficient and effective way of reducing microorganisms that can survive on surfaces (e.g., Centers for Disease Control and Prevention, 2019). Still, the frequent shifting of tools and equipment among users at the gym implies a significant risk of pathogen transmissions. The increased risk of transmission of microorganisms makes gyms and fitness centers a vital place to improve hygienic behavior, which would greatly benefit individuals, the fitness center community, and the society.

We see today, with the SARS-CoV-2 pandemic, the simple key advice to combat infectious diseases is hygienic behavior such as frequent handwashing. This is a straightforward and powerful means to reduce or avoid contagious diseases. According to Chriss (2016), several studies indicate that we frequently make choices that negatively affect our quality of life. This reflects a preference for innate behavior, the “thinking fast” vs. “thinking slow” dilemma, which may run counter to long-term rational decisions (cf. Kahneman, 2011). Simple hygiene measures in everyday life can help prevent infection-related implications for others in society. The facilitation of individual contributions in the community will thus be a prerequisite for altruistic choice behavior, improving public health (Ekström, 2012). Altruistic choice behavior is related to the “common goods” – what is done out of a concern for others’ good; in other words, for their well-being. Altruism (or social cooperation) is a conflict between short-term self-interest vs. longer-term collective interest (Van Bavel et al., 2020). Altruistic choices are shaped by our verbal community, which includes eye communications. Much of our behavior is under the control of others’ presence. The ways that society punishes or reinforce altruism often involve some kind of “eye” interaction.

Humans, and other animals, have a dedicated neural architecture for detecting facial features, including the presence of eyes (e.g., Burnham and Hare, 2007; Ernest-Jones et al., 2011). This built-in system, also known as “gaze detection,” is fast and automatic and served as a crucial evolutionary tool in ancestral environments (e.g., detecting lurking enemies and predators). Eye-like mimicry is a common anti-predator feature in nature, pointing to the strong signaling effect of glance (e.g., Stevens et al., 2008; Janzen et al., 2010). This is phylogenetically selected but is further shaped during our learning history. Humans’ social interaction depends on our ability to respond to stimuli conveyed by facial expressions and by the eyes of others, so the eyes are highly salient to humans (Vaish et al., 2017). Merely the elicited emotional responses of being watched may modify our behavior (Dear et al., 2019). The facial interpretation system is very robust. In experiments where humans are instructed not to respond to gazes, people are unable to suppress their natural response (e.g., Burnham and Hare, 2007; Frischen et al., 2007; Dear et al., 2019). Therefore, it is also possible to play the system by using images of human eyes to alter social behavior.

Several studies have addressed whether individuals’ behavior is altered by being observed by others in recent years. In fact, this may tap into the evolved “thinking-fast” responses driven by strong visual cues as the proximate cause and social acceptance as the ultimate factor. Previous research, such as Haley and Fessler (2005; generosity), Bateson et al. (2006; voluntary payment system), Ernest-Jones et al. (2011; clean-up of garbage), Ekström (2012; charitable donations), King et al. (2016; hand hygiene compliance), and Pfattheicher et al. (2018; hand hygiene compliance), illustrates that displaying images of human eyes is sufficient to alter real-life social behavior in a variety of contexts. Even small indications of observation increase individuals’ altruistic behavior (Ekström, 2012) and enhance cooperation (Sparks and Barclay, 2013). However, the literature based on both laboratory and field studies presents mixed results of artificial observation cues (e.g., Haley and Fessler, 2005; Matland and Murray, 2016; Shinohara and Yamamoto, 2018). In a meta-analysis, including 117 papers, Bradley et al. (2018) identified a small but statistically significant connection between observability and prosociality. The effect was stronger in the presence of passive observers than under conditions of perceptions of being watched. While the two meta-analysis conducted by Northover et al. (2017) found no evidence to support the claim that the “watching eyes” have effect on generosity.

Pfattheicher et al. (2018) emphasize that an individual’s behavior, such as hand washing, can be influenced by simple social nudges. According to Van Bavel et al. (2020), there is a way to leverage norms to use “nudges” in the contexts where people make choices (Mobekk and Stokke, 2020). An overall goal of nudging is to improve people’s health, happiness, and living conditions (Thaler and Sunstein, 2009). Nudge theory introduces contextual changes that, at the same time, preserve freedom of choice. Behavioral research with nudging as a method will thus help to reveal how preferences change in choice situations. Understanding how choice behavior is shaped and changed in a social environment is essential for facilitating effective action in society (Thaler and Sunstein, 2009).

Changing the context of decisions with a “simple nudge” may sway people in healthier directions toward more preferred choices. Based on previous research and the evolutionary perspective on the impact of the presence of eyes, we interpret that a picture of a pair of eyes is attention-grabbing and that the sensation of being observed will encourage a local cooperative norm – in this case, sanitizing workout equipment after use. Hence, will there exist a bidirectional link between a person’s “illusion of being watched” and the person’s hygienic behavior?

## Materials and Methods

### Study Design, Setting, and Participants

**Figure 1:**
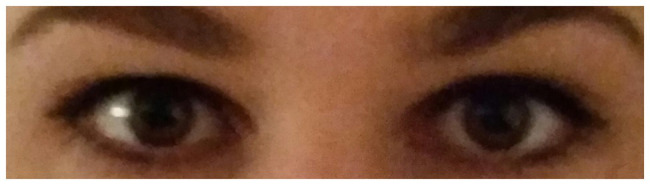
Picture of the stimulus used in the interventions in both centers, and for all sessions.

We conducted a natural field experiment where we measured if one specific social nudge – a picture of “observing eyes” (see Figure 1) – would affect the hygienic behavior of gym members. The study took place at two different gyms, Center 1 and Center 2, belonging to the same chain in Oslo, Norway. The participants in the study were gym members that attended group workout sessions at the two gyms. All observations were carried out in agreement with the center managers, and consent was given from the company to use obtained data. No personal information was collected regarding members of the gyms, and no registrations are traceable to individuals participating in the study. Ethical guidelines have been considered in all the phases of the study. The research was conducted before the SARS-CoV-2 pandemic.

The observation of the number of workout participants that cleaned the exercise equipment after use was carried out during spinning sessions at Center 1 and treadmill sessions at Center 2. The study is categorized as a “within-group design,” where the two centers function as their own control group (Cooper et al., 2007). The study’s observation phases were constructed as an ABAB design, also called a reversal design. A is a baseline phase, and B is an intervention phase. During the first phase, A, a baseline was established for the cleaning behavior (dependent variable). This is the level of responding before any intervention is introduced. Hence, the baseline phases function as control conditions. Two baseline phases (A1 and A2) and two intervention phases (B1 and B2) were conducted at both centers. The baseline phases (A1 and A2) contain choice situations in the absence of experimental manipulation. Individual behavior from session to session, if a participant happened to take part in several sessions, was not recorded.

At the beginning of the study, all information and prompts encouraging cleaning the exercise equipment after use were removed. This was also verified throughout the experiment. Before all observations, instructors were asked to hold the workout session as usual. The instructors were also informed about and reminded, not to mention cleaning routines during the workout sessions included in the study. At Center 1, four paper dispensers and nine spray bottles were available during all the observations compared to five paper dispensers and seven spray bottles at Center 2. The spray bottles and paper dispensers were in plain sight from the workout stations. All observations at both centers were performed on the same day of the week and at the same time.

**Figure 2:**
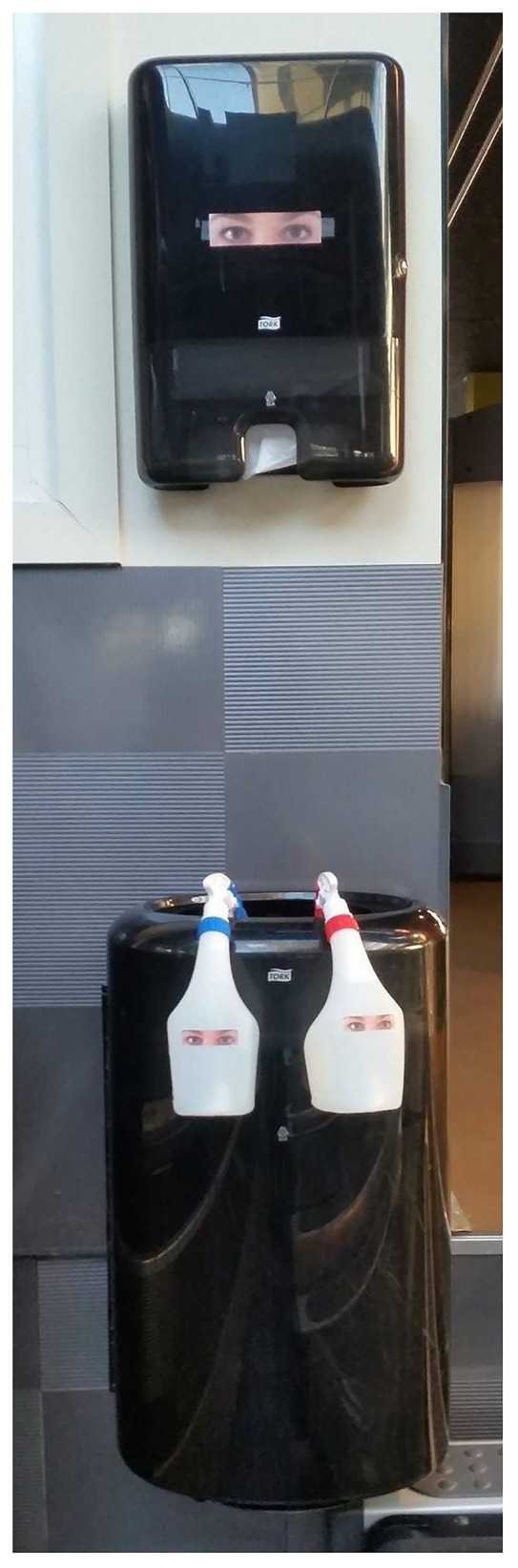
Intervention stimulus displayed on paper dispensers and spray bottles during the intervention phase. The distance between the paper dispensers and spray bottles are approximately the same in the whole fitness area and for both centers.

In the intervention phases (B1 and B2), images of “observing eyes” were attached to spray bottles and paper dispensers and were meant to function as a nudge in the choice situation (see Figure 2). The pictures were arranged visible to gym members. The cleaning equipment was located next to the exit. No gym members were in the exercise area when the pictures were attached and removed. At Center 1, the images were placed when the center opened and removed after the workout session. At Center 2, the pictures were placed in the morning and removed the next morning when the center opened. In the four observation sessions at Center 1, between 31 and 39 people were participating. At Center 2, 23 people were participating in each of the four observation sessions.

In addition to the ABAB design study, a follow-up study was carried out after 5 weeks at Center 2. The purpose was to investigate whether the effect of nudging would sustain over time since previous research of the “the watching eyes” phenomenon shows mixed results. The exercise area was prepared for this by *not* removing the images of “observing eyes” after the B2 session. Daily checks were carried out to ensure that the pictures were not damaged or altered. Five pictures were replaced throughout this period. In the follow-up study at Center 2, 23 people were participating.

### Data Recording

The dependent variable was the number of gym members choosing to use the sanitizing spray and paper dispenser to clean the exercise equipment – spinning bicycles/treadmills – after use. Data were recorded manually on a predesigned observation form by two observers. The observation form mapped the exercise area with spinning bicycles/treadmills drawn in the correct positions. When conducting the observations, the observers participated in the workout sessions, using spinning bicycles or treadmills at the back of the room. The observers washed their spinning bicycles/treadmills after all other members had left the area. In each session, the observer recorded if a position was used and whether the user cleaned the spinning bicycle/treadmill. The observations were transferred to digital representation for data analysis. The level of significance was tested by the standard Chi-square test. A limitation associated with using a Chi-square test with an ABAB design, where participants are not randomly allocated to each condition, is that we cannot guarantee that each participant only contributed data to one and only one condition. This violates one of the assumptions of the Chi-square test (McHugh, 2013).

## Results

**Figure 3:**
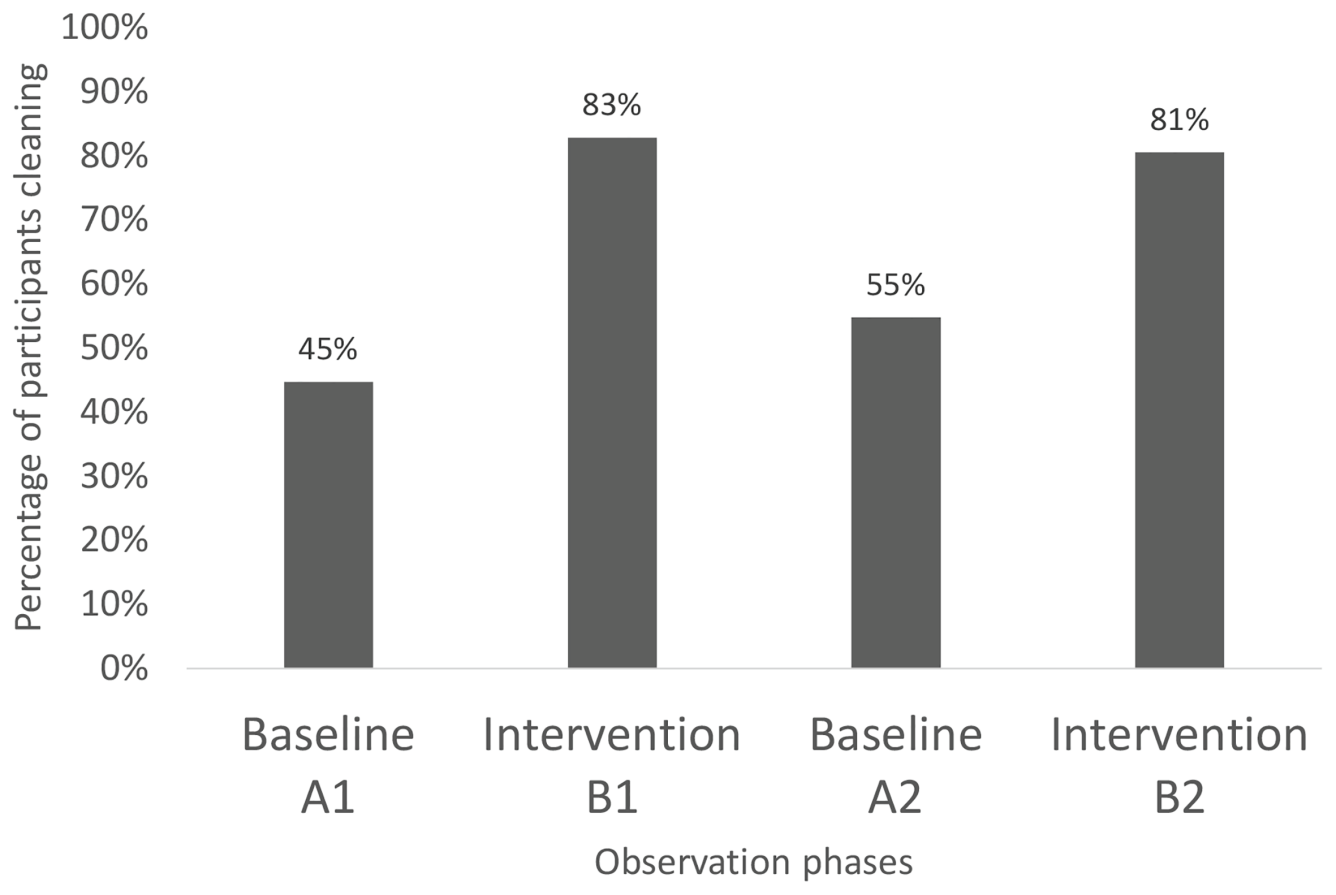
Percentage representation of cleaning behavior from Center 1 – spinning.

There were significant differences between the A (baseline) and B (intervention) phases at both centers. At Center 1, the results are based on 140 individual choice situations from two baseline phases (38 and 31 participants) and two intervention phases (35 and 36 participants). In the two baseline phases, 17 participants washed the equipment compared to 29 in the two intervention phases. In the A1 baseline phase, the hygienic behavior of one participant was not recorded due to a lack of observation by the observer. The results from the intervention phases show an increase in the number of members who washed bicycles after use, compared to the baseline phases. While targeted behavior during baseline responses ranged from 45 to 55%, the intervention increased the positive responses to 81–83% (Figure 3; Table 1). Chi-square tests revealed significant differences between baselines and interventions [*X* ^2^ (1, *N* = 140) = 16.32, *p* < 0.001] but neither within the baselines nor the interventions.

**Table 1:** Results from the spinning sessions at Center 1.

	Baseline A1	Intervention B1	Baseline A2	Intervention B2
Participants	39	35	31	36
Washed	17	29	17	29
Did not wash	21	6	14	7
Not observed	1	0	0	0

**Figure 4:**
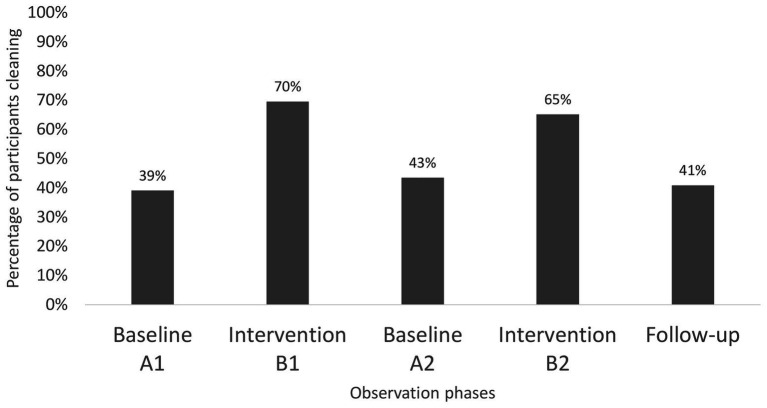
Percentage representation of cleaning behavior from Center 2 – treadmill.

At Center 2, the results are based on 92 individual choice situations from the two baseline phases and two intervention phases. In all phases, there were 23 participants. The results from the intervention phases show an increase in the number of members who washed treadmills after use, compared to the baseline phases. We found 39–41% positive baseline responses and 65–73% intervention responses (Figure 4; Table 2). Chi-square test revealed significant differences between baselines and interventions [*X* ^2^ (1, *N* = 92) = 6.31, *p* = 0.012] but neither within the baselines nor the interventions. Thus, the results were consistent with those from Center 1, yet with somewhat lower intervention responses.

**Table 2:** Results from the treadmill sessions at Center 2.

	Baseline A1	Intervention B1	Baseline A2	Intervention B2	Follow-up
Participants	23	23	23	23	23
Washed	9	16	10	15	9
Did not wash	14	7	13	8	13
Not observed	0	0	0	0	1

The results from the follow-up study, with 22 individual choice situations, did not show an increase in the number of members who washed treadmills after use compared with the baseline phases. In the follow-up phase, the hygienic behavior of one participant was not recorded due to a lack of observation by the observer. With follow-up responses of 41%, no significant difference was revealed between baseline and follow-up responses.

Interobserver agreement (IOA), calculated as a trial-by-trial IOA (Cooper et al., 2007), was between 94 and 100% in all eight observation sessions.

## Discussion

Simple hygiene measures in everyday life can help prevent infection-related consequences for others in society. Unfortunately, many people fail to engage in public health behaviors, like handwashing, which can spread infectious diseases. Finding strategies and solutions for overcoming these blunders is crucial for the health and well-being of people. A focus on individual actions and altruistic and prosocial behavior in society will thus facilitate better public health and well-being. With the SARS-CoV-2 experience, this has shown to be more critical than ever. The purpose of this study was to investigate whether a specific social nudge can improve hygienic behavior and further contribute to better health. In the present study, we build on past research showing that a picture of observing eyes, or the sensation of being watched, increases socially desirable and anticipated behavior. We wanted to investigate if a picture of observing eyes could increase the use of sanitizer and hygienic behavior among gym members. The data show that more people sanitized their workout equipment during the intervention phases than the baseline phases.

We used a reversal design, an ABAB design. One challenge with this design is that if the dependent variable changes after the intervention are introduced, it is possible that an extraneous variable is responsible for the change in the dependent variable. But, if the dependent variable changes with the introduction of the intervention, a picture of observing eyes – and then changes back with the removal of the stimuli, increases the reliability that the intervention is the cause of the behavior change. In other words, the reversal greatly increases the study’s internal validity, which was the case in this experiment. This supports the hypothesis that images of eyes prompt more prosocial behavior and that people behave altruistically (Oda et al., 2011). Another critical issue that the data revealed was that under half of the members cleaned the workout equipment in the baseline phases.

Our study confirms the positive and immediate response to “being watched.” Further, the findings extend previous research on eye images in joint responsibility of hygienic behavior such as the studies by King et al. (2016) and Pfattheicher et al. (2018). As the follow-up study revealed, this is not a long-lasting trigger but a transient effect in support of Sparks and Barclay (2013). There might be several reasons for the absence of long-term effects, such as habituation as exposure to the stimulus increases and/or social influence by other gym members’ hygienic behavior.

The results of Bateson et al. (2013) support our findings, where the phenomenon that the “eyes” seek to influence is individuals’ contribution to shared responsibility. Their findings show that images of eyes induce more prosocial behavior, independent of local norms. Ekström (2012) reveals the effect of the “eyes” on people’s generosity in a field study, while Haley and Fessler (2005) show a similar effect on generosity in a laboratory study. The “eyes” effect on people’s conscience/ethical attitudes is shown in Nettle et al. (2012), where bicycle theft decreased in areas where images of “observing eyes” were introduced. On the other hand, an increase in bicycle theft was recorded in other areas nearby, suggesting a limited effect of the intervention. The results of Nettle et al. (2012) indicate that more people adhere to ethical and moral guidelines when the illusion of being observed is introduced. However, it suggests that rule-governed behavior does not change, as the propensity to steal is maintained outside the observed range. The ultimate driver linking eyes to prosocial behavior is presumably reputation (e.g., Feinberg et al., 2012; Exley, 2018) since gossip originates from observations, and reputation has bearings on fitness.

Consequences of our actions can occur immediately or after some time. The value of delayed outcomes is often weakened over time (Critchfield and Kollins, 2001). Also, consequences that are highly likely to occur are given more value compared to more uncertain outcomes (Green and Myerson, 2004). Another issue is that people also often display an “optimism bias,” which means that bad things are more likely to befall others than oneself (Van Bavel et al., 2020). This has implications for health issues, such as infection risk, since the probability of getting an infection may be considered low. In addition, there is a delay between the time of contagion and when you become sick. At the point of choice, the cost of the seemingly trivial but unpleasant and time-consuming cleaning of the exercise equipment is higher than the risk of sanctions by the other gym members or the chance of getting an infection. This can also be viewed as a social dilemma of maximizing one’s immediate well-being or maximizing the well-being of a group of people (Rachlin, 2016). By influencing people in the point of choice with nudging, we can bridge the gap between immediate sub-optimal decisions and more optimal long-term outcomes. Small significant effects can have profound cumulative effects on our health and well-being when there is a lot at stake, such as transmitting contagious diseases.

Knowing how different nudges affect us in both the short‐ and long-term is the key to change behavior and to create new and better habits in the long run. This suggests that other means are needed to maintain the desired effect, e.g., flickering eyes, shifting cues, or other not static nudges. The challenge, though, is what happens when the novelty wears off? Maybe what we need to build new and better hygienic habits is an interdisciplinary approach combining nudging strategies with more traditional economic incentives and regulations.

### Strengths and Limitations

The results of this research were generated outside of the laboratory. Field experiments give added value since it documents naturally occurring behavior. The underlying idea behind most field experiments is to use randomization in an environment that captures the important characteristics of the real world (List and Reiley, 2010). This provides greater confidence that the results obtained are not merely an artifact of experimentation. In a natural field experiment, the subjects do not know that they are in an experiment (Harrison and List, 2004). The gym members do not know that they are participants in an experiment; this minimizes the challenges of experimentally confounding effects. Since this is a natural field experiment conducted over time, including several conditions, no manipulation checks were included since this could have influenced the next condition.

Experimental control is challenging in field experiments. The advantage is high ecological validity, but there is no random allocation of participants, and extraneous variables can influence the results. For instance, we cannot rule out that some participants were influenced by other gym members’ hygienic behavior and not by the “observing eyes.” Decisions are made by individuals who are shaped by and implanted in social environments. This means that humans, as social species, are highly sensitive to others’ influence and follow the norms of the group, especially when their reputation is at stake (Vaish et al., 2017). People tend to behave differently – are more willing to cooperate – publicly than anonymously (Oda et al., 2011).

To increase the study’s reliability, we used an ABAB design – two measures in both baselines – and intervention phases. Using an ABAB design, experimental control will be shown by the results in the different A phases (baseline) being as similar as possible and by the B phases (intervention) being as similar as possible. Our data reveal this pattern, and by including two centers, there is implicit a replication within the study. A disadvantage of using an ABAB design is that there might be a carryover effect from the B1 intervention to the A2. Most studies, especially lab studies, do not usually last long enough to study repeated behaviors and the possible decay of effects over time. The follow-up study after 5 weeks showed that the effect of the nudge has diminished.

No data about the participants were collected except for their participation and cleaning in the workout sessions. At the time of the experiment, it was not common practice at these exercise centers to register the participants. Therefore, the demographics of the participants or whether they participated in multiple sessions are not known. Introducing registration could have revealed the experiment, created questions from the participants, and added a potential bias. However, some degree of continuity can be expected, as some members of gyms have fixed exercise days and hours. Suppose the same members were present in all observations and exposed to the nudge. In that case, this might increase the confidence in the data that the intervention had an effect. Heterogeneity is a threat to internal validity (Shadish et al., 2002). To strengthen the study’s internal validity, we included two different centers with geographical distance and two different workout classes.

Since the robustness of the “watching eyes” phenomenon is still questionable, as also this study reveals when it comes to the long-term effect, further studies are needed (Oda, 2019). Behavior is context-dependent, and every intervention is unique. This requires an experimental approach to test, learn, and inform how theory translates into practice. The use of “watching eyes” is a low-cost intervention, and to some extent, it has a high impact in real-world settings, at least when there is a short time exposure. Further, carefully designed field studies, including follow-up studies and replications, are needed to draw definite conclusions of the effects of images of eyes in different situations, contexts, and populations and for how long the effect lasts.

## Data Availability Statement

The raw data supporting the conclusions of this article will be made available by the authors, without undue reservation.

## Ethics Statement

Ethical review and approval were not required for the study on human participants in accordance with the local legislation and institutional requirements. Written informed consent for participation was not required for this study in accordance with the national legislation and the institutional requirements.

## Author Contributions

HM and HJ developed the study concept and analyzed the experimental data. HJ collected the data. HM drafted the manuscript. DH, HJ, and AF contributed to the article. All authors approved the submitted version.

### Conflict of Interest

The authors declare that the research was conducted in the absence of any commercial or financial relationships that could be construed as a potential conflict of interest.
